# Ruthenium Complexes with 2-Pyridin-2-yl-1*H*-benzimidazole as Potential Antimicrobial Agents: Correlation between Chemical Properties and Anti-Biofilm Effects

**DOI:** 10.3390/ijms221810113

**Published:** 2021-09-18

**Authors:** Agnieszka Jabłońska-Wawrzycka, Patrycja Rogala, Grzegorz Czerwonka, Katarzyna Gałczyńska, Marcin Drabik, Magdalena Dańczuk

**Affiliations:** 1 Institute of Chemistry, Jan Kochanowski University of Kielce, 7 Uniwersytecka Str., 25-406 Kielce, Poland; patrycja.rogala@ujk.edu.pl; 2 Institute of Biology, Jan Kochanowski University of Kielce, 7 Uniwersytecka Str., 25-406 Kielce, Poland; gczerwonka@ujk.edu.pl (G.C.); katarzyna.galczynska@ujk.edu.pl (K.G.); 3 Institute of Physics, Jan Kochanowski University of Kielce, 7 Uniwersytecka Str., 25-406 Kielce, Poland; m.drabik@ujk.edu.pl; 4 Faculty of Environmental, Geomatic and Energy Engineering, Kielce University of Technology, 7 Tysiąclecia Państwa Polskiego Ave., 25-314 Kielce, Poland; magdar@tu.kielce.pl

**Keywords:** ruthenium complexes, electrochemistry, Hirshfeld surface analysis, antibacterial and anti-biofilm activity, contact angle, zeta potential, adhesion study

## Abstract

Antimicrobial resistance is a growing public health concern that requires urgent action. Biofilm-associated resistance to antimicrobials begins at the attachment phase and increases as the biofilms maturate. Hence, interrupting the initial binding process of bacteria to surfaces is essential to effectively prevent biofilm-associated problems. Herein, we have evaluated the antibacterial and anti-biofilm activities of three ruthenium complexes in different oxidation states with 2-pyridin-2-yl-1*H*-benzimidazole (L_1_ = 2,2′-PyBIm): [(η^6^-*p*-cymene)Ru^II^ClL_1_]PF_6_ (Ru(II) complex), *mer*-[Ru^III^Cl_3_(CH_3_CN)L_1_]·L_1_·3H_2_O (Ru(III) complex), (H_2_L_1_)_2_[Ru^III^Cl_4_(CH_3_CN)_2_]_2_[Ru^IV^Cl_4_(CH_3_CN)_2_]·2Cl·6H_2_O (Ru(III/IV) complex). The biological activity of the compounds was screened against *Escherichia coli*, *Staphylococcus aureus*, and *Pseudomonas aeruginosa* strains. The results indicated that the anti-biofilm activity of the Ru complexes at concentration of 1 mM was better than that of the ligand alone against the *P. aeruginosa* PAO1. It means that ligand, in combination with ruthenium ion, shows a synergistic effect. The effect of the Ru complexes on cell surface properties was determined by the contact angle and zeta potential values. The electric and physical properties of the microbial surface are useful tools for the examined aggregation phenomenon and disruption of the adhesion. Considering that intermolecular interactions are important and largely define the functions of compounds, we examined interactions in the crystals of the Ru complexes using the Hirshfeld surface analysis.

## 1. Introduction

Microbial resistance to antibiotics is increasingly becoming a global public health problem that threatens the successful treatment of infectious diseases. Combatting this threat is a high priority for both the European Medicines Agency and the World Health Organization. A multi-resistant strain of *Pseudomonas aeruginosa* (a Gram-negative bacterium) is one of 12 drug-resistant bacteria that requires prompt action from the scientific research community (i.e., for researchers to develop a new treatment strategy) [1]. *P. aeruginosa* was identified as the most dangerous bacterium due to its ability to survive in different environments. Drug resistance is mainly related to the mechanism of pathogenicity of microorganisms, which is the ability to form biofilms [2,3,4,5]. Overall, it is estimated that biofilms account for approximately 80% of total bacterial infections in the human body [2]. Microbial cells within biofilms are nearly 1000 times more resistant to toxic substances compared to their planktonic counterparts [3,4]. It should be emphasized that conventional antibiotic therapy is mainly effective against planktonic cells [3,4]. The strong resistance of bacterial biofilms is related to both bacteria aggregation in multicellular communities and the structure of extracellular polymeric substances (EPSs) matrix [6]. Once biofilms form, these features influence the enhancement of genetic exchange, the concentration of quorum sensing (QS) signal molecules, the development of persisters, and the chelation of antimicrobials. They also provide physical protection. The disruption of bacteria aggregation in multicellular communities and/or the destruction of the structure of the EPS matrix may result in host defenses being able to resolve infections and restore the efficacies of antibiotics. There are two ways to approach biofilms, to prevent them from forming or removing already formed biofilms. To prevent biofilm formation, the attachment of planktonic cells to surfaces or the maturation of early microcolonies to fully structured biofilms must be disrupted. It can be achieved by modifying the surface to which microbes attach or by treating microbial cells with chemical compounds to block or weaken their attachments to the surface. Here, we focused on chemical treatments for the inhibition of biofilm formation. 

Biofilm inhibition can be caused by surface conditioning chemicals or by repressing microbial adhesion molecules. The hydrophobic properties of microorganisms are some of the most important nonspecific adhesion factors that play an essential role in the multiplication of microorganisms on solid surfaces. This parameter is strongly related to the structure and integrity of the cell membrane, and variables, such as the microbial activity of bacteria, growth conditions, pH, temperature, secretion of extracellular substances, and time, may have a strong influence on it [7,8]. The surface of *P. aeruginosa* is negatively charged, and many surface structures, proteins, or lipids increase its hydrophobicity. The surface properties, characteristic of a given microbial species, are decisive for the colonization of a given surface. Strong hydrophobic properties affect the auto-aggregation of bacterial cells, which means a higher degree of adhesion. 

One alternative to microbial resistance is by using metal complexes as anti-biofilm agents. Benzimidazole-based metal complexes are attractive, in particular, due to their rich coordination chemistry. Moreover, 2-pyridin-2-yl-1*H*-benzimidazole (2,2′-PyBIm) and its derivatives are powerful chelating ligands, which are used for synthesizing complexes with intriguing biological applications. Owing to their potential biological activity, benzimidazole and its derivatives have been extensively investigated in medicine. Benzimidazole core is present in compounds that have their applications in diverse therapeutic areas, such as antihistamine [9], anti-kinase [10], anti-ulcer [11], anti-inflammatory [12], analgesic [13], antioxidant [14], anti-HIV-1 [15], as well as antibacterial [16]. Benzimidazole is a vital pharmacophore in drug discovery. Benzimidazole-based drugs, such as albendazole, carbendazim, and tilomisole, are well exemplified [17,18,19,20]. Benzimidazole is one of the heterocyclic ring system components used to treat different types of cancer. For instance, 2-substituted benzimidazoles exhibits cytotoxicity against various cancer cell lines [21,22,23]. Moreover, many bis-benzimidazoles are inhibitors of DNA topoisomerase I [24]. The interaction of benzimidazole derivatives with model proteins is well-known, and it has been investigated by using X-ray crystallography and spectrometric techniques [25]. 

This work is a continuation of our research regarding metal complexes, such as anti-biofilm agents. The utilization of ruthenium complexes in this area seems to be justified by their previously reported anticancer, antiviral, and antibacterial properties [26,27,28,29,30]. The Ru complexes with 2-pyridin-2-yl-1*H*-benzimidazole (L_1_ = 2,2′-PyBIm) formulated as: [(η^6^-*p*-cymene)Ru^II^ClL_1_]PF_6_, *mer*-[Ru^III^Cl_3_(CH_3_CN)L_1_]·L_1_·3H_2_O and (H_2_L_1_)_2_[Ru^III^Cl_4_(CH_3_CN)_2_]_2_[Ru^IV^Cl_4_(CH_3_CN)_2_]·2Cl·6H_2_O were isolated previously. Their crystal structures and physicochemical characterizations were published in [31,32]. Such full characterization was a starting point for understanding the selected physical, chemical, and biological properties of the Ru complexes. Our main aim was to estimate the effectiveness of the Ru complexes against the biofilm of a bacterial laboratory strain of PAO1 and a clinical strain of LES B58. The anti-biofilm activity was determined by a percentage (%) of inhibition biofilm formation (crystal violet technique) and epifluorescence microscopy, in order to observe the morphological changes of bacterial microcolony. To identify the factors relevant for the anti-biofilm activity of [(η^6^-*p*-cymene)Ru^II^ClL_1_]PF_6_, (Ru(II) complex), *mer*-[Ru^III^Cl_3_(CH_3_CN)L_1_]·L_1_·3H_2_O (Ru(III) complex), and (H_2_L_1_)_2_[Ru^III^Cl_4_(CH_3_CN)_2_]_2_[Ru^IV^Cl_4_(CH_3_CN)_2_]·2Cl·6H_2_O (Ru(III/IV) complex) towards *Pseudomonas aeruginosa*, we: (i) obtained the Ru complexes according to the published method [31,32] for the biological tests purpose; (ii) conducted analytical and spectroscopic characterization of the obtained complexes for their purity confirmation; (iii) conducted supplementary studies on the electrochemical properties of the title compounds; (iv) conducted the Hirshfeld surface analysis to visualize intermolecular interactions in the Ru complexes. We quantitatively evaluated *P. aeruginosa* PAO1 adhesion in the presence of the Ru complexes. We also investigated the impact of the Ru complexes on the parameters of bacterial surface, such as potential (ζ) and the contact angle. These measurements allowed us to determine the change of wettability and hydrophobicity of a bacterial cell modified by the Ru complexes. Additionally, antibacterial activity against *Escherichia coli*, *Staphylococcus aureus*, and *Pseudomonas aeruginosa* was screened. Arguably, many organic compounds and metal complexes have general toxicity, unrelated to microbes, but related to normal human cells. To demonstrate the safe use of the complexes selected, their toxicity was assessed by measuring cell viability against the human non-tumorigenic lung epithelial cell line (BEAS-2B).

## 2. Results and Discussion

**Figure 1:**
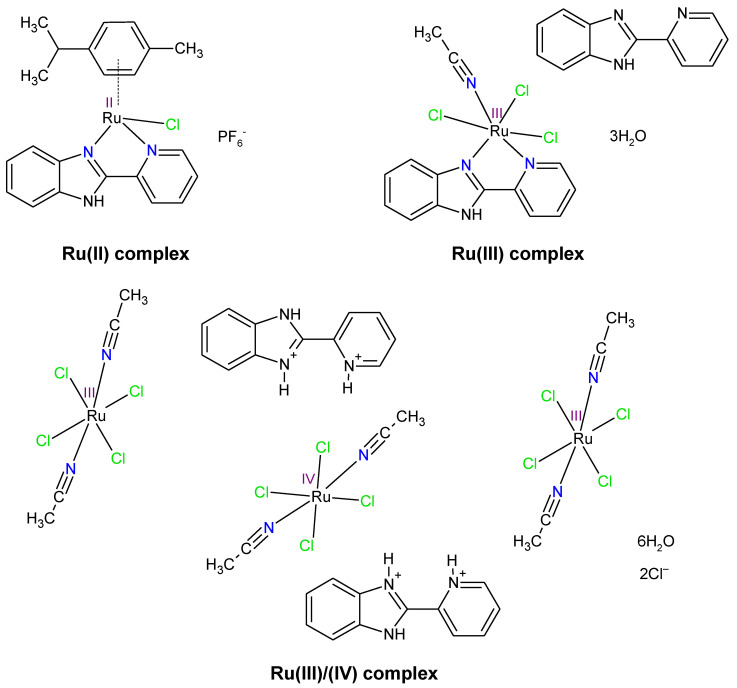
The structures of the ruthenium complexes in different oxidation states with 2-pyridin-2-yl-1H-benzimidazole.

The coordination compounds were synthesized following the procedure described in the literature [31,32], using the mother solution (0.1 M RuCl_3_) or the Ru(II) precursor ([(η^6^-*p*-cymene)Ru(μ-Cl)Cl]_2_), the N,N-donor ligand (2,2′-PyBIm, L_1_), and proper solvents as starting materials. To confirm the structures of the ruthenium complexes, the IR spectra were recorded, and the elemental analysis was carried out (see supplementary materials). The structure and properties of the Ru complexes are in agreement with published data [31,32]. The isolated Ru complexes were in +II ([(η^6^-*p*-cymene)Ru^II^ClL_1_]PF_6_), +III (*mer*-[Ru^III^Cl_3_(CH_3_CN)L_1_]·L_1_·3H_2_O), and mixed +III and +IV oxidation states ((H_2_L_1_)_2_[Ru^III^Cl_4_(CH_3_CN)_2_]_2_[Ru^IV^Cl_4_(CH_3_CN)_2_]·2Cl·6H_2_O) (Figure 1). Figure 1 illustrates that the Ru(III/IV) complex structure displays three metal centers—two in +III oxidation state and one in +IV oxidation state. It was observed that 2,2′-PyBIm acted as a chelating ligand in the Ru(II) and the Ru(III) complexes [31,32]. However, the two protonated forms of the 2,2′-PyBIm in the structure of the Ru(III/IV) complex were present. The Ru(III) and Ru(III/IV) complexes were six-coordinated with distorted octahedral geometry. In turn, the Ru(II) complex belonged to the half-sandwich class of organometallic compounds that possessed four-legged piano-stool geometry. In the case of the Ru(III/IV) complex, physicochemical characterization was also completed with a magnetic study. The obtained effective magnetic moment value was 3.66 μ_B_. It corresponds to four unpaired electrons in the system. The presence of one unpaired electron is associated with two Ru^3+^ centers. Meanwhile, two unpaired electrons are attributed to Ru^4+^ center. These results are in agreement with the crystal structure reported in [31]. The isolated products were used following the electrochemical (cyclic voltammetry (CV) and differential pulse voltammetry (DPV)) and intermolecular atomic contacts (HS analysis), the biological (MIC determination, crystal violet technique, epifluorescence microscopy, MTS assay), and bacterial cell surface studies (zeta potential, contact angle).

Stability in solution of the metal complexes is crucial in the in vitro screening studies. The solution stability of the ruthenium complexes presented was analyzed by absorption UV-Vis spectrophotometry. For this purpose, the Ru(II) and Ru(IV) complexes were dissolved in H_2_O, and the Ru(III) complex was dissolved in H_2_O/DMSO (49/1 *v*/*v*). Their UV-Vis spectra were recorded directly after dissolution, as well as after 24 h of incubation at 37 °C. The results obtained showed that the Ru complexes are stable in solvents at the time provided for the biological experiments (24 h). The relevant bands were not shifted. Only slight changes in the intensity of the absorption maxima were observed. (Figure S1). 

### 2.1. Hirshfeld Surface Analysis of Ru Complexes in Different Oxidation States

Intermolecular interactions in the Ru complexes were further investigated and visualized by Hirshfeld surfaces (HS). The HS analysis has become a useful tool for explaining the nature of intermolecular interactions that affect the packing of molecules in crystals [33]. Over the last decade, the HS analysis has rapidly gained popularity. It can be employed to visualize and quantify various non-covalent interactions that stabilize the crystal packing [34,35]. This applies to the identification of close contacts, in particular, which are deemed important.

In the case of the Ru(II) complex, the HS analysis was published in [32], and concerned only one of two metal centers. Due to the presence of two symmetrically-independent Ru(II) centers in the structure [32], the HS analysis was repeated (concerning two metal centers), and the compiled results, differing from that published previously, were included in this work. Size, shape, and flexibility are important topological parameters that describe the functional specificity and different types of interactions in compounds. The asymmetry in the shape of molecules is investigated among others using the asphericity parameter (Ω), calculated from the eigenvalues of the moment of inertia tensor. The asphericity parameters for the Ru complexes, however, were different from one another. The Ω parameter almost doubled its value when going from the Ru(III) complex to the Ru(III/IV) complex, having a value of 0.127 (Ω ^1/2^ = 0.36) for the Ru(III) complex and 0.239 (Ω ^1/2^ = 0.49) for the Ru(III/IV) complex. Such values indicate that the shape of the surfaces is closer to the oblate object. It can be observed that the Ω parameter increased in the following order for the ruthenium complexes: the Ru(III) complex (0.127) < the Ru(II) complex (0.138) < the Ru(III/IV) complex (0.239). In turn, the globularity parameter (G) accounted for the deviation of the surface area from a sphere of the same volume, being equal to unity for a sphere. In comparison to another quantitative parameter, the G parameter for the complexes was very similar, in the range from 0.631 to 0.658. These values indicate that the surface of the ruthenium complexes was rather closer to a spherical shape.

**Figure 2:**
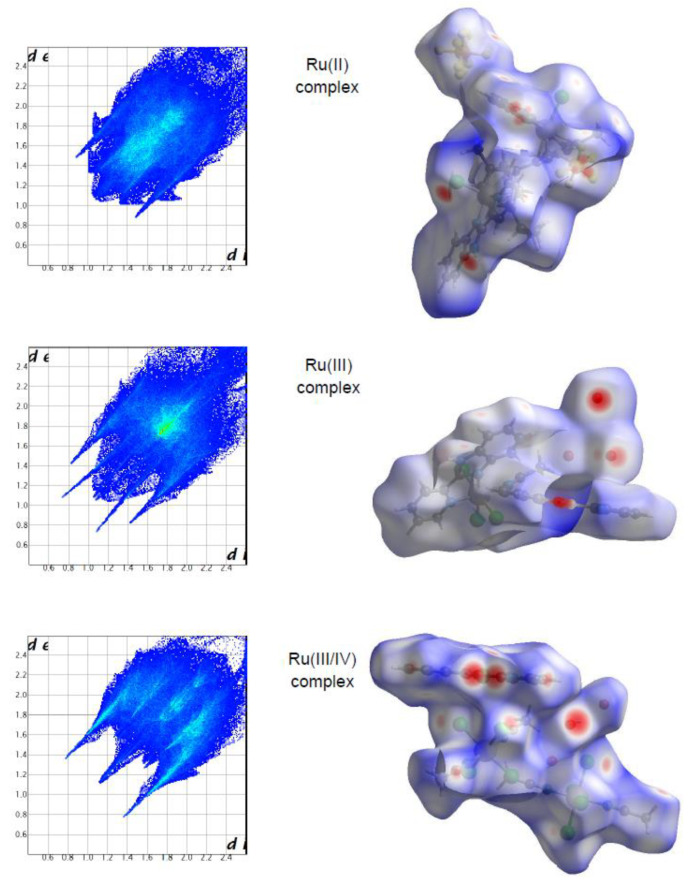
Two-dimensional fingerprint plots for all intermolecular contacts in the Ru complexes. Surfaces to the right highlight the relevant *d*_norm_ surface patches associated with the specific contacts.

The molecular Hirshfeld surface of the ruthenium complexes is presented in Figure 2. Three-dimensional (3D) Hirshfeld surface maps were obtained using red–white–blue *d*_norm_ surface maps (surface resolution −0.3 to 3.3 Å), where red indicates shorter contacts with negative *d*_norm_ values, white indicates close van der Waals contacts with zero *d*_norm_ values, and blue indicates longer contacts with positive *d*_norm_ values. Figure 2 shows that the Hirshfeld surface analysis of the Ru(III) complex has fewer types of close contacts (red spots) in comparison to the Ru(II) complex, and next, the Ru(III/IV) complex. The 2D fingerprint plots obtained by the Hirshfeld surface analysis were also studied. The overall fingerprint plots for the Ru complexes are shown in Figure 2. The main reciprocal intermolecular interactions (X⋅⋅⋅H, H⋅⋅⋅H, C⋅⋅⋅H, O⋅⋅⋅H, Cl⋅⋅⋅O, where X = Cl, F) were obtained using the 2D fingerprint plot and the 3D *d*_norm_ surfaces of the complexes.

**Figure 3:**
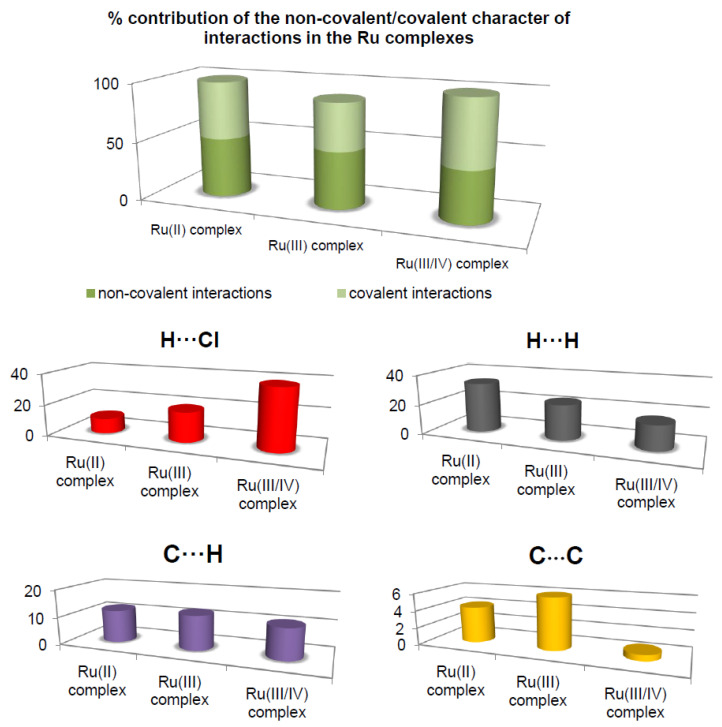
Comparison of the percentage of contribution of important contacts for the ruthenium complexes.

The most prominent type of contacts in the Ru(III) complex corresponded to H···H contacts; they contributed 23.4% to the overall surface contacts (Figure 3). The C···H/H···C contacts with a 12.4% proportion appeared as pair of widely scattered wings in the 2D fingerprint plot. The O···H/H···O contacts (Figure 3) contributed 14.9% to the HS and formed a pair of sharp and long spikes. The Cl···H/H···Cl contacts, contributing 19% to the overall surface, were also depicted as spikes. For the Ru(II) complex, the H···F/F···H contacts were dominant (33.9%), and made contribution to the Hirshfeld surface as pair of spikes. The H···H interactions contributed in similar proportion to the total Hirshfeld surface (33.3%). Another significant spots in the Hirshfeld surfaces corresponded to reciprocal C···H and H···Cl contacts (11.8 and 9.8% respectively). For the Ru(III/IV) complex, the Cl···H/H···Cl (38.3%) surface contacts dominated the fingerprint profile. The H···H interactions contributed second most (16.3%), to the total HS (Figure 3) as the spike. The pair of the wide wings represents the reciprocal C···H contacts with a contribution of 11.0%. The Cl···O/O···Cl contacts were viewed as pair of spikes contributing 9.0% to the HS.

The contributions of other contacts to the Hirshfeld surface were small or negligible and amounted to 0.1–6.2% for all three structures. As can be seen, the common feature of all the complexes is the presence of the three repeating contacts—H⋅⋅⋅H, Cl⋅⋅⋅H, and C⋅⋅⋅H. However, they occurred in the complexes at different percentages. A noticeable difference is that each complex had an additional interaction not found in the others (H⋅⋅⋅O, H⋅⋅⋅F, and Cl⋅⋅⋅O). Importantly, the analysis of % contribution showed that the polar character of interactions in the Ru(III/IV) complex (56.1%) predominated significantly, in comparison to that of the Ru(II) and the Ru(III) complexes, where those interactions reached a lower percentage (48.6% and 40.1%, respectively; Figure 3).

### 2.2. Electrochemical Studies

The role of electrochemical techniques cannot be underestimated in an analysis of synthetic products due to their relationship with bioactive properties. Many of the essential physiological processes are based on redox chains involving enzyme-catalyzed processes. There is a set of similarities between electrochemical and biological reactions concerning electron transfer (ET) pathways [36]. Thus, there are many examples where electrochemistry, dealing with different aspects of electron transfer (ET), contributes significantly to medicinal chemistry [37]. Although electrochemical parameters do not give absolute correlation with biological activity data, regarding the enormous complexity of biomedical chemistry, such techniques are still a very useful tool [37]. 

**Figure 4:**
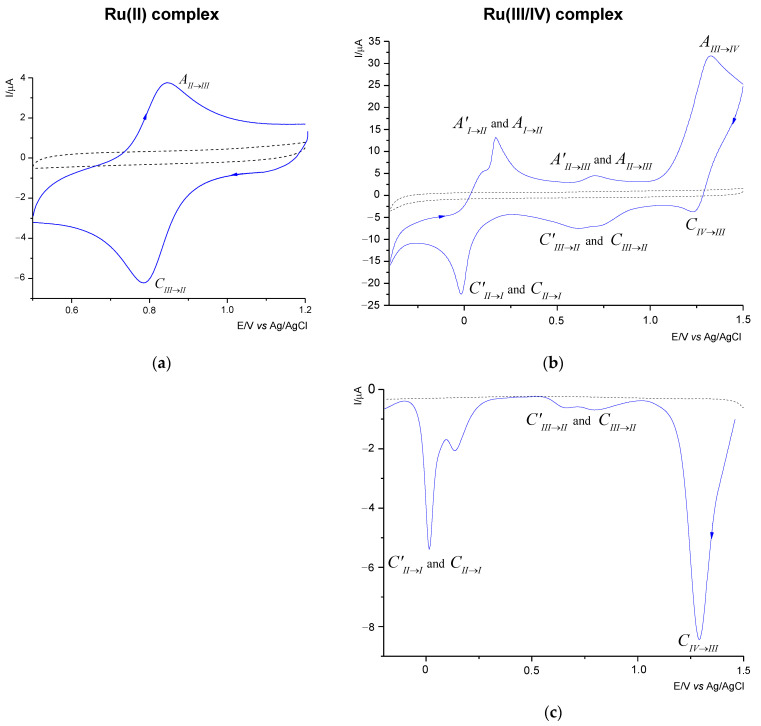
CV (**a**,**b**) and DPV (**c**) curves recorded in acetonitrile containing 0.1 M TBAPF_6_ and 1 mM Ru(II) complex/Ru(III/IV) complex (—) or 1 mM ligands (---), (CV conditions: GCE, Ø = 2 mm, scan rate 100 mV/s, T = 25 °C; DPV conditions: CF, Ø = 33 µm, pulse amplitude 20 mV, pulse width 80 ms, scan rate 20 mV/s).

The electrochemical behavior of the Ru(II) and the Ru(III/IV) compounds was investigated in the AN/0.1 M TBAPF_6_ solvent/electrolyte system through cyclic voltammetry (CV) and differential pulse voltammetry (DPV). The CV curves were recorded from the initial potential −0.85 V (for the Ru(III/IV) complex) and 0.4 V (for the Ru(III) complex) vs. Ag/AgCl (1M NaCl). The cyclic voltammograms of 1.0 mM solutions of each complex in the AN were collected to probe the effect of the 2,2′-PyBIm on the reduction potentials of the complexes, and the impact of the ligand exchange processes. To verify the reversibility of the redox couples and the number of the electrons exchanged, the CV diagnostic criteria, Δ*E*_p_ = *E*_pa_ − *E*_pc_ (*E*_pa_, *E*_pc_ denote the potentials of the anodic and cathodic peaks, respectively) were applied [38]. The scan rates of 50, 100, and 200 mV/s were also chosen for those experiments to estimate the reversibility or irreversibility of an electrode process. The electrochemical study of the Ru(III) complex was previously reported in [31]. The voltammetric curves of the Ru(II) and the Ru(III/IV) complexes are shown in Figure 4. Table 1 summarizes the voltammetric data of the compounds investigated. As shown in Figure 4, the ligand (---2,2′-PyBIm) appeared not to be electroactive in the solvent system chosen.

The Ru(II) complex shows only one redox pair. The voltammetric response of the Ru(II) complex (Figure 4a) proved the cathodic process to be attributable to the Ru(III)→Ru(II) reduction (Table 1). The reduction process corresponded to the oxidation process the Ru(II)→Ru(III), as evidenced by the peak at 0.841 V for the scan rate of 100 mV/s. The value of the peak-to-peak separation indicates that the process is *quasi*-reversible with the exchange of one electron. The nature of the electrode reaction was confirmed by slight changes in the Δ*E*_p_ values with the increase of the scanning speed (Table 1).

By comparison with the Ru(II) complex, we ascribed more redox pairs to the Ru(III) and the Ru(III/IV) complexes. As in the case of the Ru(III) complex [31] and the mixed-valence Ru(III)−Ru(IV) species, we ascribed three redox pairs (Figure 4b). The Ru(III/IV) complex underwent three reduction processes; the first one at 1.230 V, the second one at 0.735 and 0.616 V, and the third one at approximately −0.016 V respectively. As some of the peaks were poorly shaped and wide, the DPV technique was employed. It allowed us to separate the adjoining peaks. Figure 4c, shows two pairs of the separated signals, at 0.795 and 0.665 V, and 0.0135 and 0.015 V, respectively.

The analysis of the DPV curve (Figure 4c) revealed that in the two redox processes, two maxima were observed (Table 1) related to the presence of the ruthenium complexes in different oxidation states. On the return sweep (Figure 4b), three signals were observed, in the range from −0.85 to 1.5 V, and attributed to oxidation processes (Table 1). As previously observed for reduction, the anodic peaks were wide and splitting in two cases. The three observed pairs in the CV curve are attributed to the reduction and oxidation processes of the Ru(IV)↔Ru(III), the Ru(III)↔Ru(II), and the Ru(II)↔Ru(I), respectively. The CV diagnostic criteria exceeded the theoretical value of 0.058 V for a reversible one-electron redox couple. It was found that the Δ*E*_p_ values increased significantly with the scan rates, which gives evidence for the irreversible nature of the three systems. Moreover, the recorded cathodic signals, as in the case of the anode signals, shifted with the increase of the scan rate, which is also a characteristic feature of irreversible processes. An interesting feature of the Ru(II)/Ru(III) system was noticed by comparing the position of the *E*_pc_ and the *E*_pa_ peaks in the three investigated complexes. Namely, they occurred in similar potential ranges but showed slight shifts to each other. This may be due to the difference in the Ru(III/IV) coordination environment compared to the Ru(II) and the Ru(III) complexes.

### 2.3. Inhibition of Planktonic Bacterial Growth by Ruthenium Complexes

The bacteriostatic activity of the ruthenium complexes in different oxidation states, along with the starting compounds used for their synthesis (RuCl_3_·xH_2_O, Ru(II) precursor, 2,2′-PyBIm), were evaluated by determining their minimum inhibitory concentration (MIC). The MIC parameter is used as an important research tool to determine the in vitro activity of new antimicrobial agents, and data from such studies can be used to determine MIC breakpoints. The antibacterial efficacy of the compounds tested was compared with a commercial drug—streptomycin. The results are summarized in Table 2. 

When all the strains were incubated in the presence of the Ru(III) simple salt or the ruthenium(II) precursor, no relevant effects against either control strains were observed. In turn, fairly moderate effectiveness was exhibited by the ligand, which acted at the highest concentration tested towards *S. aureus* and *E. coli*. The ruthenium complexes had different activity profiles across the strains. As shown in Table 2, the most effective compound against all the strains tested in this study was the Ru(III) complex (MIC = 347 μg/mL or 693 μg/mL). The Ru(II) complex inhibited the growth of three of the four bacteria at a concentration of 1 mM (611 μg/mL). Meanwhile, the Ru(III/IV) complex exhibited the bacteriostatic activity against one strain. Taking into account the resistance of the *Pseudomonas* strain, an important observation is that all investigated Ru complexes were active against the *P. aeruginosa* PAO1 with MIC values equal to or greater than 0.5 mM. The greatest inhibitory action against this pathogen was detected for the Ru(III) complex. The effect of different concentrations (0.0625–1 mM) of the Ru(III) complex on cell growth of the *P. aeruginosa* PAO1 is shown in Figure S2. The concentration of 0.5 mM of the Ru(III) complex reduced the cell growth up to 72% in comparison with the control. A similar activity profile to the tested Ru compounds against *P. aeruginosa* was observed for the Fe(III) complex with 2,2′-PyBIm as the chelating ligand [39]. In turn, some studied Ir(III) and Re(I) complexes with the N,N-pyridylbenzimidazole derivatives turned out to be ineffective against *P. aeruginosa* at the highest tested concentration (>32 μg/mL) [40,41]. On this basis, the antibacterial activity of the ruthenium complexes can be assessed as good or moderate.

### 2.4. Effect of Ru Complexes on Hydrophilic Bacterial Surface Properties

**Figure 5:**
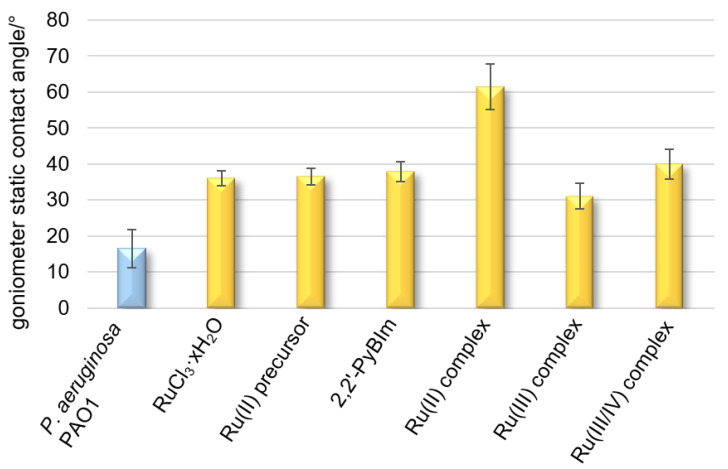
Wettability (contact angle) of *P. aeruginosa* PAO1 with original surface and with the addition of the Ru complexes.

Hydrophobicity is the driving force behind the adhesion of bacterial cells to solid surfaces when the distance from a given surface is large. It also promotes the self-aggregation of microorganisms. Hydrophobicity/hydrophilicity, as an essential property of a surface, is defined as the affinity of a given structure to polar solvents (in this case water). Contact angle measurements of liquid droplets on surfaces of bacterial cells are used to characterize surface properties modified to provide information on the evolution of hydrophilic/hydrophobic character induced by the different functionalizations or the presence of different substances. The contact angle for the surface of the cells closely adjacent to each other, forming a uniform layer, is 16° (Figure 5). The low contact angle value results from the high hydrophilic character of the bacterial surface, as a consequence of the high density of the polar groups on it. The bacterial film treated with the Ru complexes reached a contact angle in the range of 31–61° (Figure 5), denoting a moderate or strong hydrophobic surface. The following trend in hydrophobicity is observed for the Ru complexes examined: the Ru(III) complex < the Ru(III/IV) complex < the Ru(II) complex. On the contrary, for the tightly adhering bacterial layer treated with ruthenium(III) chloride, *p*-cymene, or 2,2′-PyBIm, the contact angles were in the very narrow range of 36–38°. 

### 2.5. Effect of Ru Complexes on the Value of Electrokinetic Potential (Zeta Potential) of Bacterial Cells 

Knowledge of the electrical properties of planktonic cells is very useful to combat biofilms formed by pathogenic bacteria. The change in the value of the electrokinetic potential may indicate physical changes taking place in a cell and may be a useful diagnostic tool characterizing the phenotypic features of a given bacterial species [42,43]. Due to the specific structure of cell walls related to the presence of characteristic functional groups derived from lipopolysaccharides, teichoic acids, or peptidoglycan, bacteria are endowed with a negative surface charge. This charge determines the formation of the electric bilayer and the formation of the zeta potential (ζ). These parameters are closely related to the adhesion and colonization abilities of bacteria to various surfaces, both biotic and abiotic. The following step of the research focused on estimating the surface charge of bacterial cells by measuring the zeta potential. Possible changes in the zeta potential as a result of treating bacterial cells with the Ru complexes would explain the phenomenon of bacterial cell aggregation and adhesion disruption of these. They are caused mainly by the charge present on the surface of bacterial cells. However, an important role is played by the previously discussed hydrophobicity and composition of the cell wall, properties of solid surfaces, and many parameters related to the reaction environment, e.g., ionic strength, the concentration of buffer solutions, and pH value. Therefore, the presence of an electric charge on the surface of microorganisms has a direct effect on aggregation and adhesion to solid surfaces. The zeta potential is characterized by an electrical double layer on the cell surface, which influences the electrophoretic mobility of bacteria and their tendency to aggregate.

**Figure 6:**
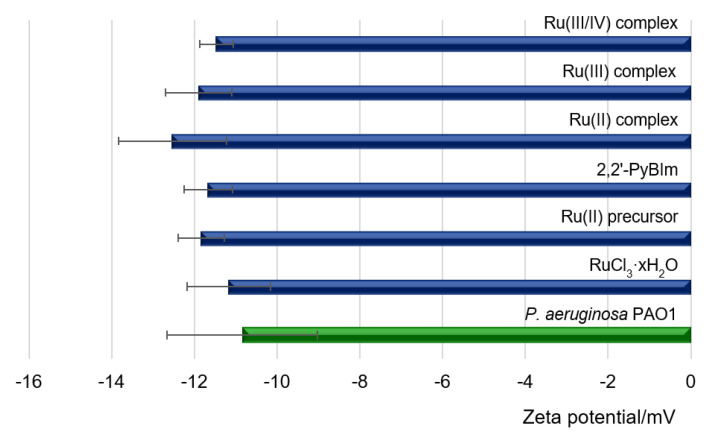
Zeta potentials of *P. aeruginosa* PAO1 with original surface and with the addition of the Ru complexes.

In this study, the measurements of the zeta potential of cells were carried out at pH = 7.3 ± 0.2 for the control sample, which was a bacterial suspension in the medium and for bacterial cells exposed to the Ru complexes. The measurement results are presented in Figure 6. The ζ value of −11 mV was recorded for free bacterial cells. The negative electrostatic charge on the cell surface in Gram-negative bacteria resulted from the exposure of phosphoric and carboxylate groups on the cell surface, associated with membrane lipopolysaccharides [44]. Compared to bacterial cells treated with the Ru complexes, the measured zeta potentials remained at a similar level (Figure 6). 

### 2.6. Effects of Test Compounds on Biofilm Formation

**Figure 7:**
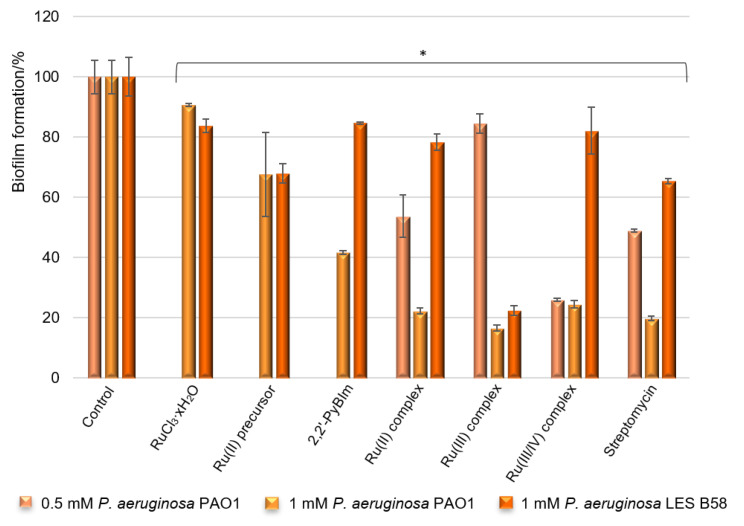
*P. aeruginosa* PAO1 and LES B58 biofilms formation in the presence of RuCl_3_·xH_2_O, Ru(II) precursor, free ligand, and Ru complexes (concentrations of compounds—0.5 and 1 mM). The absorbance of the control was considered to represent 100% of biofilm formation (results were considered significant when compared to control; * *p* < 0.05. Data are presented as mean ± SD, *n* = 4).

As *P. aeruginosa* is a highly opportunistic human pathogen, it can colonize various sites, causing serious clinical consequences. The ability of the bacteria to biofilm formation, associated with the production of different extracellular virulence factors, allow bacteria to adapt to environmental changes and host defense mechanisms. To determine the capacity of potential antimicrobial agents to prevent the formation of biofilms, a crystal violet assay is frequently used. The crystal violet staining is one of the basic tests performed to obtain quantitative information about the relative density of cells adhering to the material. With this in mind, the effects of the ruthenium complexes on biofilm formation by laboratory and clinical isolates of *P. aeruginosa* were analyzed, in terms of total biomass and cellular metabolic activity, by epifluorescence microscopy. The results of the biological activity of the compounds against the *P. aeruginosa* PAO1 and LES B58 are presented in Figure 7. 

According to the results of the tests, the ruthenium complexes showed a significant reduction in the *P. aeruginosa* PAO1 biofilm formation. The Ru compounds were able to inhibit the biofilm formation, reducing the biomass by 83% (the Ru(III) complex), 78% (the Ru(II) complex), and 75% (the Ru(III/IV) complex) when the concentration was 1 mM. Thus, the activity of the compounds is comparable at this concentration. It is worth noting that these values are similar to those obtained for the reference standard being evaluated—streptomycin. The results also demonstrated that the free ligand (2,2′-PyBIm) exhibited moderate effectiveness at the tested concentration, and was less active than its ruthenium complexes. Decreasing the concentration of the added ruthenium compounds (0.5 mM) prompted a lower level of reduction in the biofilm formation of the *P. aeruginosa* PAO1 strain in the case of the Ru(II) and the Ru(III) complexes. These complexes were able to inhibit the biofilm formation, reducing the biomass by 46% and 15% at the tested concentration, respectively. Interestingly, more than 70% of the biofilm biomass formed by the PAO1 was inhibited at both 0.5 mM and 1 mM concentrations of the Ru(III/IV) complex (Figure 7). The activity of the ruthenium complexes was varied against the clinical isolate strain of the *P. aeruginosa* LES B58 biofilm. Most noteworthy is the Ru(III) complex at 1 mM, which reduced 77% of the *P. aeruginosa* LES B58 biofilm.

**Figure 8:**
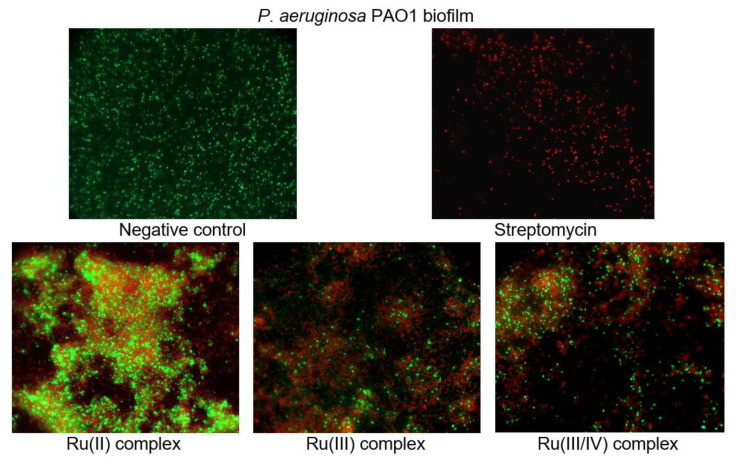
Epifluorescence microscopy images of *P. aeruginosa* PAO1 biofilm treated with 1 mM of ruthenium complexes. Biofilm was stained with nucleic acid stains using the FilmTracer™ LIVE/DEAD Biofilm Viability Kit (live cells are represented by the color green; dead cells are represented by the color red). The epifluorescence microscopy images were captured at 1000× magnification.

To investigate the cell viability and morphological changes of the *P. aeruginosa* PAO1 biofilm treated with the ruthenium complexes, the LIVE/DEAD biofilm viability kit (Invitrogen) was used, and fluorescence microscope observations were performed. The LIVE/DEAD assay staining solution is a mixture of two fluorescent dyes (SYTO-9 and propidium iodide) that differentially label live and dead cells. The live cell dye, SYTO-9, penetrates bacterial membranes freely and fluoresces when bound to DNA, staining intact, viable cells green. Propidium iodide enters only the cells with compromised membranes and stains the dead cells, generating red fluorescence. Figure 8 shows the effect of the ruthenium complexes on the *P. aeruginosa* PAO1 biofilm architecture. As can be seen in the representative images, the cells are well spread and viable on the control surface, unlike the surfaces treated with the ruthenium complexes. The Ru compounds led to morphological and structural damage of the *P. aeruginosa* PAO1 biofilm. Their presence in the culture caused the aggregation of cells and led to the death of many bacterial cells. 

### 2.7. Quantitative Study of P. aeruginosa PAO1 Adhesion in the Presence of Ru Complexes

**Figure 9:**
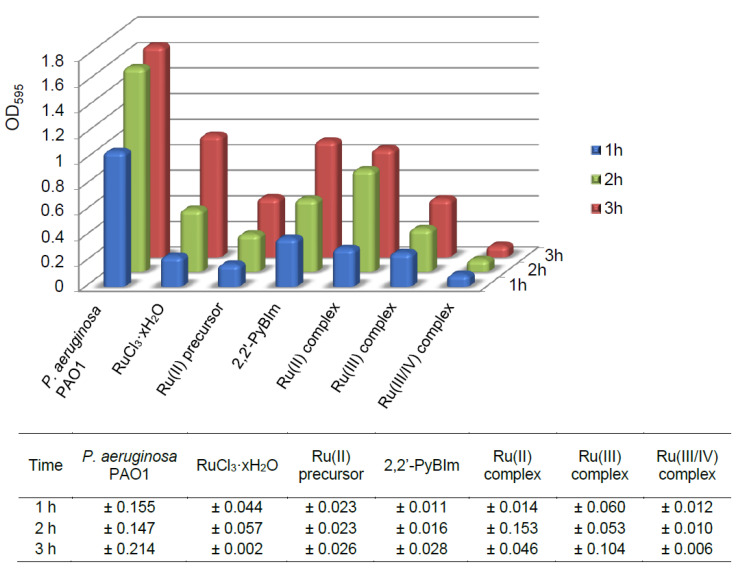
Monitoring early stage of *P. aeruginosa* PAO1 adhesion in the presence of the Ru complexes (after 1, 2, and 3 h) through quantitative analysis. The standard deviation are also presented.

As is known, the adhesive properties of bacteria play an important role in the early stages of biofilm development. Upon irreversible attachment of bacterial cells to the surface, biofilm is formed in which its extracellular matrix helps maintain structural integrity. In this experiment, we examined the effect of the Ru complexes on the adhesion process of the *P. aeruginosa* PAO1 over time, after 1, 2, and 3 h. The degree of bacterial adhesion was assessed spectrophotometrically, using crystal violet as staining reagent. The sub-inhibitory concentrations of the compounds were used: 0.5 mM for the Ru(III) complex, and 1 mM for the Ru(II) and the Ru(III/IV) complexes. The results of the tests are presented in the Figure 9 and Table S1. The lowest cell adhesion of the P. aeruginosa PAO1 was observed after the Ru(III/IV) complex exposure. In comparison to the control, this compound resulted in the adhesion of 7% (average value) only. The Ru(II) and the Ru(III) complexes promoted higher initial cell adhesion in the P. aeruginosa PAO1 strain. For the Ru(III) complex, the percentage of the adhesion ranged from 21% to 28%. In turn, for the Ru(II) complex, the adhesion was definitely the highest—for the first hour of the experiment, it was 28%, and for the following two hours, it reached the values of 50% and 51%, respectively.

### 2.8. Cytotoxicity Activity

The cytotoxicity is an important indicator for toxicity evaluation of substances. The half-maximal inhibitory concentration (IC_50_) is the most widely used and informative measure of a potential biological agent’s efficacy. The metabolic viability assay of the human non-tumorigenic lung epithelial cell line (BEAS-2B were purchased from the American Type Tissue Culture Collection (ATCC, Rockville, MD)) treated with the ruthenium complexes was performed by the MTS test. The results were expressed as IC_50_ values. The Ru complexes exhibited different cytotoxic activities. Namely, the Ru(II) complex was cytotoxic to non-tumorigenic epithelial cell line from human bronchial epithelium at the concentration of 250 µM. In turn, the IC_50_ value of the Ru(III) complex was 1000 µM.

### 2.9. Correlation and Regularity between Chemical Properties and Anti-Biofilm Effects

All tested ruthenium complexes show a high level of inhibition of the *Pseudomonas aeruginosa* PAO1 biofilm formation (75–83%), which proves their high efficiency at 1 mM concentration. Based on these results, the following order of anti-biofilm activity can be assigned: the Ru(III/IV) complex < the Ru(II) complex < the Ru(III) complex. The analyzed data show that the free ligand—2,2′-PyBIm displays a moderate activity, and in combination with ruthenium ions, it gives a synergistic effect against the *P. aeruginosa* PAO1 strain. The % of biofilm inhibition of the Ru(II) and Ru(III) complexes decreases with the concentration decreasing—0.5 mM. Whereas, the Ru(III/IV) complex activity remains at the same level regardless of the concentration used (74%). The Ru(II) complex is characterized by anti-biofilm activity amount of 46% (at concentration of 0.5 mM) despite the fact that arene-ruthenium complexes containing *p*-cymene ring are described in the literature as compounds with a very high antimicrobial and anticancer activities [45,46,47,48]. The lowest percent inhibition of biofilm formation is observed in the presence of the Ru(III) complex. Remarkably, a row of the anti-biofilm activity is reversed at concentration of 0.5 mM used. It should be noted that the Ru(III/IV) complex has a different structure compared to those described above (Figure 1). Namely, the ligand (2,2′-PyBIm) in the Ru(III/IV) complex does not form a coordination compound with the ruthenium ion. The protonated form of the ligand is in the crystal lattice and is balanced by chloride ions. Such significant differences are reflected in the anti-biofilm activity of the Ru complexes. Nevertheless, the presence of ruthenium in the +III and +IV oxidation states in the coordination environment of four chloride ions and two acetonitrile molecules results in a high degree of inhibition of biofilm formation. Interestingly, it turns out that among the investigated complexes, the best effect is shown by the anionic complex compared to complexes with a neutral or positive charge. These results are in accordance with the data obtained by epifluorescence microscopy. The presence of the Ru compounds in the culture contributed to damages to the bacterial cell walls, and ultimately caused the death of many cells. The ruthenium complexes tested were also found to be more active against the *P. aeruginosa* PAO1 biofilm than against the planktonic form. Taking the mean minimum inhibitory concentration (MIC) for the *P. aeruginosa* PAO1 strain, the order of the planktonic growth inhibition by the Ru complexes was identical to that of the biofilm (at 1 mM).

The HS analysis revealed some correlation between biological activity and an important topological parameter, asphericity, which can affect it. It can be observed that the Ω parameter increases in the following order for the ruthenium complexes: the Ru(III) complex < the Ru(II) complex < the Ru(III/IV) complex. This row shows a positive correlation with biological activity. As the asphericity parameter increases, the anti-biofilm activity increases (at 0.5 mM). In contrary, as the asphericity parameter increases, the anti-biofilm activity decreases (at 1 mM). Moreover, it can be demonstrated that the predominance of the polar character of the intermolecular contacts also correlates with the anti-biofilm activity of the ruthenium complexes (Table 3). The higher contribution of the polar character of contacts, the higher the anti-biofilm activity of the ruthenium complexes (at 0.5 mM). As observed for the concentration of 1 mM, the higher contribution of the polar character of contacts, the lower the anti-biofilm activity of the Ru complexes. In the case of the interactions possessing a low difference of electronegativity between the pair contacts, such dependency on biological activity was not observed. The interesting feature of the analysis is associated with the interactions such as C⋅⋅⋅H and C⋅⋅⋅C (Figure 3), with a low difference of electronegativity between the pair contacts. Although C⋅⋅⋅C contacts are interpreted as minor (range of 0.7–6.2%), they are deemed important for biological activity. By contrast, it was observed that the percentage of contribution of both C⋅⋅⋅H and C⋅⋅⋅C interactions increased in the following order for the Ru complexes: the Ru(III/IV) complex < the Ru(II) complex < the Ru(III) complex. The row is inversely consistent with that of the anti-biofilm activity (at 0.5 mM).

Some regularity between the biological activity and the electrochemical properties of the ruthenium compounds was also found. An interesting feature of the Ru(II)/Ru(III) system was noticed by comparing the position of the *E*_pc_ and the *E*_pa_ peaks in the three investigated complexes. Namely, they occurred in similar potential ranges but showed slight shifts to each other. This may be due to the difference in the structures of the Ru complexes. However, we noticed a trend for increasing oxidizing properties for the Ru complexes regarding the Ru(II)/Ru(III) redox pair. Namely, the electrochemical properties (E_1/2_) increased in the following order: the Ru(III/IV) complex < the Ru(II) complex < the Ru(III) complex (Table 3). The correlation of the E_1/2_ with the % biofilm inhibition showed a decreasing tendency—the higher the half-wave potential, the lower the biological activity (at 0.5 mM). 

The addition of the ruthenium complexes to the bacteria increased the contact angle of the bacterial layer. This means changing the properties of the bacterial outer membrane from hydrophilic to hydrophobic. Given the LPS structure (lipopolysaccharide) and its amphiphilic character, and the presence of numerous phosphoryl, amine, carbonyl, and carboxylate groups, therein, we should expect a relatively polar surface with a low contact angle for the bacterial surface. We can assume the hydrophobic effect imparted by the ruthenium complexes contributes to diluting the polar contribution of the terminal groups of the LPS structure. It is likely due to the involvement of the terminal polar groups in strong intermolecular interaction, through hydrogen bonding, between the Ru complexes and a bacterial surface. The results evidence a significant modification of the surface character of the PAO1 cells towards hydrophobic features as a result of their exposure to the Ru complexes. At the same time, it was found that bacterial surface hydrophobicity increased to varying degrees in the presence of the Ru complexes (Table 3). This phenomenon is consistent with the earlier reports that bacterial adhesion to the surface can be reinforced by changes in bacterial surface hydrophobicity. Moreover, the previous papers also reported that a higher degree of bacterial surface hydrophobicity was associated with higher bacterial adhesiveness [49,50]. Two other studies reported that bacterial surface hydrophobicity was an important factor promoting biofilm formation and the binding of antimicrobial agents [51,52]. Contrary to the changes in cell surface hydrophobicity, the zeta potential value, after the Ru complexes exposure, did not differ from the value for untreated cells. Interestingly, not in every case, the increase in hydrophobicity quantitatively influenced the increase in the adhesion in the experiment (Table 3). It seems that this correlation was met for the Ru(II) complex, in which the increase in hydrophobicity was the greatest, and the adhesion was as high as 51%. It is interesting that the increase in hydrophobicity for the Ru(III/IV) complex practically inhibited the adhesion. Meanwhile, for the Ru(III) complex, the smallest of the specified increases in hydrophobicity caused adhesion at the level of 28%. 

Therefore, the increase in hydrophobicity led to the formation of large aggregates, which was clearly observed in the epifluorescence images for all the complexes. Nevertheless, the increase in hydrophobicity reduced the dispersion of microcolonies to the aquatic environment. This means a disruption in the process of spreading biofilm, and as a result, a reduction in the production of biofilm by *P. aeruginosa.*

## 3. Materials and Methods

RuCl_3_·xH_2_O, 2-pyridin-2-yl-1*H*-benzimidazole, [(η^6^-*p*-cymene)Ru(μ-Cl)Cl]_2_ were purchased from Sigma Aldrich (St. Louis, MO, USA) and used as received. The solvents—concentrated hydrochloric acid, methanol, and acetonitrile—were sourced from commercial vendors and used without further purification. Ethanol was acquired from Linegal Chemicals (Warsaw, Poland) and was purified by using a distillation method. The starting (mother) ruthenium(III) chloride solution (0.1 M) was prepared according to the procedure described in the literature [31].

### 3.1. Chemical Experiments

#### 3.1.1. Syntheses of Ruthenium Complexes in Different Oxidation States

The coordination compounds were synthesized following a procedure described in the literature [31,32] using the mother solution (0.1 M RuCl_3_) or Ru(II) precursor ([(η^6^-*p*-cymene)Ru(μ-Cl)Cl]_2_), 2,2′-PyBIm and proper solvents as a starting materials.

#### 3.1.2. Physical Measurements

The IR spectra were recorded on a Nicolet 380 FT-IR spectrophotometer in the spectral range 4000–500 cm^−1^ using the ATR-diffusive reflection method. Magnetic measurements were carried out on a magnetic susceptibility balance (Sherwood Scientific) at room temperature by Gouy’s method, using Hg[Co(NCS)_4_] as a calibrant. The data were corrected for diamagnetic contributions, which were estimated from Pascal’s constants. The solubility of the complexes was tested in solvents such as water, methanol, ethanol, dimethyl sulfoxide, N,N-dimethylformamide, acetone, and acetonitrile. The complexes were found to be well soluble in water, alcohols, acetonitrile, and dimethyl sulfoxide (DMSO). The stability of the Ru complexes in aqueous solution (the Ru(II) and Ru(III/IV) complexes) or in an aqueous solution with the addition of DMSO (the Ru(III) complex) was checked by UV-Vis spectrophotometry. The concentrations of prepared solutions of the compounds were 1 mM. The final concentration of DMSO did not exceed 2% by volume, analogously to biological tests. The absorption spectra were measured on a V-630 UV-Vis spectrophotometer from Jasco. The spectra of the compounds were recorded after dissolution, as well as after 24 h of incubation at 37 °C.

#### 3.1.3. Hirshfeld Surface Calculations

HS analysis has become a very useful tool for explaining the nature of intermolecular interactions that affect the packing of molecules in crystals. Molecular Hirshfeld surfaces calculations were performed using the Crystal Explorer package ver. 3.1 [53]. When the *.cif file of the title compounds was entered into the Crystal Explorer program, all of the bond lengths to hydrogen were automatically modified to the standard neutron values (CH = 1.083 A). 

The Hirshfeld surface of a molecule was mapped using the descriptor *d_norm_*, which encompassed two factors: *d_i_*, representing the distance of any surface point nearest to the internal atoms, and *d_e_*, representing the distance of the surface point nearest to the exterior atoms. The calculated *d_norm_* was a normalized contact distance, which was defined in terms of *d_i_*, *d_e_*, and the van der Waals (vdW) radii of the atoms [35].

$$d_{norm\;}=\;\frac{d_{i}-r_{i}^{vdW}}{r_{i}^{vdW}}+\;\frac{d_{e}-r_{e}^{vdW}}{r_{e}^{vdW}}$$

The value of *d_norm_* was negative or positive when intermolecular contacts were shorter or longer than r vdW, respectively. The *d_norm_* values were mapped onto the Hirshfeld surfaces using a red–blue–white color scheme as follows: red regions represented closer contacts and a negative *d_norm_* value; blue regions represented longer contacts and a positive *d_norm_* value; and white regions represented the distance of contacts equal to exactly the vdW separation with a *d_norm_* value of zero [35]. In turn, the shape index is highly sensitive to very subtle changes in the surface shape. The information delivered by the shape index were consistent with 2D fingerprint plots. The whole fingerprint region and all interactions were a combination of *d_e_* and *d_i_*. The surfaces were made to be transparent to allow visualization of the molecular moiety in a similar orientation for all of the structures around which they were calculated. The 2D fingerprint plots were produced using the standard 0.4–2.8 Å and view with the de and di distance scales displayed on the graph axes. The distance from the Hirshfeld surface to the nearest nucleus inside and outside the surface has been marked by *d_i_* and *d_e_*, respectively.

#### 3.1.4. Electrochemical Measurements (CV, DPV)

Voltammetric experiments were performed using a Model M161E electrochemical analyzer connected with Model M162 preamplifier (mtm-anko, Cracow, Poland) and controlled via a Pentium computer using mEALab 2.1 software (mtm-anko, Cracow, Poland). The details of the procedure have been described previously [54]. Electrochemical investigations of the ruthenium complexes and free ligand were performed in a CH_3_CN containing 1 mM compound with 0.1 M tetrabutylammonium hexafluorophosphate (TBAPF_6_) (from Fluka, electrochemical grade) as a supporting electrolyte. The electrochemical properties of the Ru complexes were studied by cyclic voltammetry (CV) on glassy carbon electrode (GCE) (2 mm in diameter A = 0.0314 cm^2^ (Mineral, Warsaw)). Some experiments were performed with the use of differential pulse voltammetry (DPV) on carbon fiber (CF) disk microelectrode (33 µm in diameter (BASi, United Kingdom)). DPV voltammograms were registered using a pulse amplitude of 20 mV, pulse width of 80 ms and scan rate of 20 mV s^−1^. This technique is considered a convenient method because of its good sensitivity selectivity and resolution of the signals, limited influence of adsorption phenomena on recorded curves and thus excellent reproducibility [55]. 

### 3.2. Biological Experiments

#### 3.2.1. Bacterial Strains and Cultivation

The in vitro antimicrobial activity of the ligand and its ruthenium complexes were evaluated against representative Gram-positive (*Staphylococcus aureus* ATCC 6538P) and Gram-negative (*Escherichia coli* ATCC 8739, *Pseudomonas aeruginosa* PAO1 (biofilm model strain) and *Pseudomonas aeruginosa* LES B58 (clinical isolate)) bacteria. The *P. aeruginosa* PAO1 and LES B58 isolates were derived from the International *Pseudomonas aeruginosa* Reference Panel. Panel is available from the Belgian Co-ordinated Collection of Microorganisms (BCCM)/LMG Bacteria Collection, Ghent University, Gent, Belgium (http://bccm.belspo.be/about-us/bccm-lmg, accessed on 30 July 2021).

The bacterial strains studied were cultivated in Trypticase Soy Broth (TSB) medium (Biocorp, Warsaw, Poland) for 18 h at 37 °C with shaking (160 rpm). Overnight cultures of bacteria were diluted 1:100 into fresh TSB medium. Cultures as a source of bacteria were used the following the biological tests.

#### 3.2.2. Minimal Inhibitory Concentration

A broth microdilution method was used to determine the minimum inhibitory concentrations of the tested samples of the compounds. The Ru(II) and Ru(III/IV) complexes, the ligand, and the precursors were prepared by dissolving compounds in distilled water. Meanwhile, the Ru(III) complex was dissolved in distilled water with addition dimethyl sulfoxide (2%). The stock concentrations of tested compounds were 2 mM. The serial two-fold dilutions were made in a concentration range from 1 mM to 0.0625 mM in the sterile 96-well microtiter transparent plates (Greiner, Monroe, NC, USA) containing nutrient broth. After that, diluted suspensions were added to appropriate wells. The inoculated plates were incubated at 37 °C for 24 h. The negative control (bacterial culture in the medium) and positive control (antibiotic control—streptomycin) were used as references to determine the growth inhibition of bacteria. The MIC parameter was recorded as the lowest concentration of the compound at which the isolate was completely inhibited (as evidenced by the absence of visible bacterial growth). The experiments were performed using the Infinite M200 PRO microplate reader (Tecan, Männedorf, Switzerland). Tests were conducted as three independent repeats.

#### 3.2.3. Zeta Potential and Contact Angle Measurements

The electrokinetic potential (zeta potential, ζ) and contact angle were measured using *P. aeruginosa* PAO1 cultures in TSB medium. Bacterial cell surface was modified by the overnight culture in the presence of tested compounds (1 mM solution of the Ru(II) and the Ru(III/IV) complexes; 0.5 mM solution of the Ru(III) complex). The cultures modified by the Ru complexes were washed with 0.9% sterile saline in triplicate. Next, cells were resuspended in TSB medium to a density of the 0.5 McFarland standard [50]. The measurements of the electrokinetic potential were performed on the Zetasizer Nano-ZS analyzer (Malvern Instruments, Malvern, UK) with using Laser Doppler Velocimetry (LDV) method. In order to determine the electrokinetic potential of cells, the suspension of modified microorganisms was introduced into a measuring DTS1070 cuvette (750 μL) with two electrodes (Malvern Instruments, Malvern, UK), which was then placed in the Zetasizer apparatus. The instrument uses laser light scattering in a microtube under electrophoresis conditions to determine the electrophoretic mobility of the particles. The electrokinetic potential of samples was measured at constant pH = 7.3 ± 0.2 and determined from electrophoretic mobility using the Smoluchowski equation [56,57]. Measurements were performed as three independent repeats. 

For hydrophobicity tests, overnight cultures modified by Ru complexes, were washed in triplicate (4000× *g*, 5 min), and resuspended in deionized water, and a sample was placed on a glass microscope slide. Slides were dried at 37 °C for 1 h, for water evaporation. Bacterial cells then formed a dense and uniform layer (thin film), which was suitable for contact angle measurements. Crystal violet staining was performed on additional slides to be sure that thin film closely covered the slide surface. The hydrophobicity was analyzed using the contact angle goniometer (OCA 15EC, DataPhysics Instruments, Filderstadt, Germany) and dynamic sessile drop. Moreover, 2 μL of distilled water was dropped onto the dry bacterial cell surface using a micro-syringe, and the contact angles were measured. At least five readings of contact angles were taken to obtain the average value for each Ru complex. Hydrophobicity was determined by % based on literature criteria [58]. 

#### 3.2.4. Inhibition of Biofilm Formation and Quantitative Study of P. aeruginosa Adhesion

The overnight cultures treated with the Ru complexes were exposed to biofilm formation assay and adhesion. The inhibition effect of the tested compounds on biofilm formation by the *P. aeruginosa* PAO1 and LES B58 strains was measured by crystal violet method using 96-well microtiter plates [59]. The amount of biofilm formed was determined as described previously [54]. Stock solutions of the Ru compounds (2 mM) were prepared in distilled water or in distilled water with the addition of dimethyl sulfoxide (the concentration of DMSO did not exceed 2% by volume). The final concentrations of compounds in the cell cultures were in the range 0.0625–1 mM. Additionally, fresh medium was used as a negative control and a streptomycin as a positive control. Absorbance of the eluted crystal violet was measured on an Infinite M200 PRO microplate reader at wavelength of 595 nm (Tecan, Männedorf, Switzerland). The assays were performed at least in three independent experiments.

Adhesion was estimated by quantitative analysis of the *P. aeruginosa* PAO1 cells adhered to 96-well transparent flat-bottomed microtiter plates (Greiner, Monroe, NC, USA), using crystal violet staining. The overnight culture was transferred (200 μL) to the new 96-well microtiter plates and incubated for 1, 2, and 3 h. Next, the planktonic cells were washed away with distilled water by immersing whole plate. The plate and its contents were dried out, and then 200 μL of the crystal violet solution (0.1%, *w/v*) was added, and next incubated for 15 min in room temperature (RT). The crystal violet was removed and the plate was washed with distilled water again, in triplicate. The crystal violet was extracted from adhered cells by filling the wells by 96% ethanol (incubation 15 min. in RT). The plate was subjected to optical density (OD) measurement (λ = 595 nm) in Infinite M200PRO microplate reader (Tecan, Männedorf, Switzerland). 

The measurement results, expressed in absorbance or optical density (OD) units, were converted into percentages to allow the comparison of numerical data obtained in different experiments.

#### 3.2.5. Live/Dead Staining of the Bacterial Biofilm

Fluorescence microscopy was used to image live/dead cells in the *P. aeruginosa* PAO1 biofilm. First, the *P. aeruginosa* PAO1 biofilm was cultivated in 6-well microtiter plates on glass coverslips (22 × 22 mm, Menzel Gläser, Germany) in TSB medium, at 37 °C for 24 h without shaking. Then, the culture was supplemented with solutions of the ruthenium complexes (1 mM). After 24-h incubation, the coverslips were carefully washed with sterile water in order to remove non-adherent cells. Microcolonies formed on the glass surface were stained with a FilmTracer™ LIVE/DEAD^®^ Biofilm Viability Kit (Invitrogen, Carlsbad, CA, USA) according to the manufacturer’s protocol. After 15 min incubation at room temperature in the dark, the samples were washed with water to remove the excess dyes. Images were collected with a ZEISS Axio Scope.A1 epifluorescence microscope. The experiments were repeated three times to obtain consistent results.

#### 3.2.6. Cytotoxicity Activity (MTS Test)

The human non-tumorigenic lung epithelial cell line (BEAS-2B cells were purchased from the American Type Tissue Culture Collection (ATCC, Rockville, MD, USA)) was cultured at 37 °C in a humidified 5% CO_2_ atmosphere in plastic dishes in LHC-9 serum-free bronchial epithelial growth medium on non-coated plates. The cytotoxic properties of the ruthenium complexes were measured by MTS Cell Proliferation Assay Kit (Abcam, ab197010) in accordance with the manufacturer’s instructions. The cells were seeded into a 96-well plate and incubated with the ruthenium complexes in the concentration range of 15–1000 µM for 24 h at 37 °C in a humidified atmosphere of 5% CO_2_. After incubation, a solution of MTS (3-(4,5-dimethylthiazol-2-yl)-5-(3-carboxymethoxyphenyl)-2-(4-sulfophenyl)-2H-tetrazolium) was added to each well and incubation at 37 °C for 4 h. The measurement of absorbance of the solution related to the number of live cells was conducted on a TECAN Spark Microplate Reader (TECAN, Männedorf, Switzerland) at 490 nm. All samples were tested in three independent experiments. The results were normalized to the control. Inhibitory concentrations (IC_50_) represent the concentrations of the tested samples required to inhibit 50% cell proliferation; they were calculated from the mean values of data from the wells.

Due to the aftermath reaction and formation of crystals after using the MTS solution, the measurement reading for the Ru(III/IV) complex was inconclusive.

#### 3.2.7. Statistical analysis 

Statistical analysis was performed using one-way analysis of variance (ANOVA). Significance was set at *p* < 0.05.

## 4. Conclusions

The presented work is a step forward in the direction of extending the knowledge related to a new strategy for *P. aeruginosa* PAO1 biofilm inhibition. Our results indicate that the Ru complexes with 2,2′-PyBIm are good candidates for the development of new anti-biofilm agents. The enhancement of the anti-biofilm activity of 2,2′-PyBIm, in combination with Ru ions, indicates a beneficial effect of this procedure (synergistic effect). Moreover, the data show that the anti-biofilm activity of the Ru(II) and Ru(III) complexes decrease with decreasing value of the concentration used. In contrast, anti-biofilm activity of the Ru(III/IV) complex remains at the similar level. As can be seen for the oxidation states, no correlation with anti-biofilm activity was found.

The presented anti-biofilm effectiveness of the Ru complexes results from the inhibition of biofilm formation trough their effect on adhesion. The bacterial surface hydrophobicity increased to varying degrees in the presence of the Ru complexes. Surprisingly, the increase in hydrophobicity for the Ru(III/IV) complex practically inhibited the adhesion. Meanwhile, for the Ru(III) complex, the smallest of the specified increases in hydrophobicity, caused adhesion at the level of 28%. It likely results from other accompanied mechanisms of action of the tested compounds. As expected, for the Ru(II) complex, the increase in hydrophobicity was the greatest, and the greatest % of adhesion was accompanied with it. It can be seen clearly that the Ru complexes have modified the surface character of the PAO1 cells. Functional groups in the ligand structures have likely produced the various possible interactions with biological targets in the bacterial surface. The HS analysis indicates that some types of contacts in the crystals of the Ru complexes could possibly affect anti-biofilm activity. As a result, the increase in hydrophobicity led to the formation of large cell aggregates (it was associated with higher bacterial adhesiveness). As expected, modification of the surface character of the *P. aeruginosa* PAO1 cells by the Ru complexes disrupted the adhesion. Consequently, the increase in hydrophobicity reduces the dispersion of microcolonies to the aquatic environment. This means a disruption in the process of spreading biofilm, and as a result, a reduction in the production of biofilm by *P. aeruginosa*. In this manuscript, some correlations between structural, electrochemical properties, and biological effects were successfully shown. The above aspects indicate the legitimacy of the study undertaken. However, some aspects still require clarification and need additional specialist research.

## Figures and Tables

**Table 1:** Electrochemical data (in V vs. Ag/AgCl) for the ruthenium complexes obtained by cyclic voltammetry (CV) on GCE and by differential pulse voltammetry (DPV) on the CF disk microelectrode.

**Complex**	**Scan rate** **mV/s**	**Ru(IV)↔Ru(III)**			**Ru(III)↔Ru(II)**			**Ru(II)↔Ru(I)**			**Method**	**Lit.**
		***E*** **_pa_**	***E*** **_pc_**	**Δ*E*_p_**	***E*** **_pa_**	***E*** **_pc_**	**Δ*E*_p_**	***E*** **_pa_**	***E*** **_pc_**	**Δ*E*_p_**		this work
**Ru(II)** **complex**	50				0.837	0.787	0.050				CV	
	100				0.841	0.785	0.056					
	200				0.848	0.778	0.070					
		Ru(IV)↔Ru(III)			Ru(III)↔Ru(II)			Ru(II)↔Ru(I)				[31]
		*E*_pa_	*E*_pc_	Δ*E*_p_	*E*_pa_	*E*_pc_	Δ*E*_p_	*E*_pa_	*E*_pc_	Δ*E*_p_		
**Ru(III)** **complex**	50	1.145	~1.060	~0.08	0.880	0.827	0.053	−0.214	−0.288	0.074	CV	
	100	1.155	~1.050	~0.10	0.882	0.825	0.057	−0.209	−0.290	0.081		
	200	1.160	~1.040	~0.12	0.885	0.822	0.063	−0.200	−0.290	0.090		
		Ru2(IV)↔Ru2(III)			Ru2(III)↔Ru2(II) and Ru1(III)↔Ru1(II)			Ru2(II)↔Ru2(I) and Ru1(II)↔Ru1(I)				this work
		*E*_pa_	*E*_pc_	Δ*E*_p_	*E*_pa_	*E*_pc_	Δ*E*_p_	*E*_pa_	*E*_pc_	Δ*E*_p_		
**Ru(III)/(IV)** **complex**	50	1.322	1.228	0.094	0.694	0.804	0.110	0.156	lack of signal	-	CV	
						0.706	0.012	0.100	0.022	0.078		
	100	1.326	1.230	0.096	0.704	0.735	0.031	0.170	lack of signal	-		
						0.616	0.088	0.102	−0.016	0.118		
	200	1.332	1.234	0.98	0.726	0.702	0.024	0.192	lack of signal	-		
						0.590	0.136	0.122	−0.056	0.178		
	20		1.290			0.795			0.135		DPV	
						0.665			0.015			

Δ*E*_p_ = |*E*_pa_ − *E*_pc_|.

**Table 2:** Results of the minimum inhibitory concentration (MIC) for the compounds evaluated, expressed in mM and μg/mL.

Compound	BACTERIA							
	*S. aureus*		*E. coli*		*P. aeruginosa* PAO1		*P. aeruginosa* LES B58	
	mM	μg/mL	mM	μg/mL	mM	μg/mL	mM	μg/mL
RuCl_3_·xH_2_O	>1	>207	>1	>207	>1	>207	>1	>207
Ru(II) precursor	>1	>612	>1	>612	>1	>612	>1	>612
2,2′-PyBIm	1	195	1	195	>1	>195	>1	>195
Ru(II) complex	1	611	1	611	1	611	>1	>611
Ru(III) complex	0.5	347	0.5	347	0.5	347	1	693
Ru(III/IV) complex	>1	>1546	>1	>1546	1	1546	>1	>1546
Streptomycin	0.0625	36	0.125	73	0.0625	36	0.5	291

**Table 3:** Comparison of physicochemical properties (scan rate 100 mV) with biological effects for the Ru complexes.

Parameter		Ru(II) Complex	Ru(III) Complex	Ru(III/IV) Complex
E_1/2_ (V); type of the process	Ru(II)↔Ru(I)	---	−0.250; *Q* [31]	---, 0.043; *I*
	Ru(III)↔Ru(II)	0.813; *Q*	0.854; *R* [31]	0.720, 0.660; *I*
	Ru(IV)↔Ru(III)	---	~1.10; *I* [31]	1.278; *I*
Anti-biofilm activity (0.5 mM) (%)		46	15	74
Hydrophobicity (%)		68	34	44
Adhesion after 3 h (%)		51	28	6

Type of the process: *R*—reversible, *Q*—*quasi*-reversible, *I*—irreversible.

## Data Availability

Data sharing not applicable.

## Supplementary Materials

The following are available online at https://www.mdpi.com/article/10.3390/ijms221810113/s1.
