# Supplementary material for: Ruthenium Complexes with 2-Pyridin-2-yl-1H-benzimidazole as Potential Antimicrobial Agents: Correlation between Chemical Properties and Anti-Biofilm Effects
Source: Int J Mol Sci. 2021 Sep 18;22(18):10113. doi: 10.3390/ijms221810113 (PMC8471145; doi:10.3390/ijms221810113)
Supplement: Supplementary file 1 [file ijms-22-10113-s001.zip › ijms-1342690-supplementary.pdf]

*Electronic Supplementary Information*

# Ruthenium Complexes with 2-Pyridin-2-yl-1*H*-benzimidazole as Potential Antimicrobial Agents: Correlation Between Chemical Properties and Anti-biofilm Effects

Agnieszka Jabłońska-Wawrzycka[*1], Patrycja Rogala[1], Grzegorz Czerwonka[2], Katarzyna Gałczyńska[2], Marcin Drabik[3], Magdalena Dańczuk[4]

[1] *Institute of Chemistry, Jan Kochanowski University of Kielce, 7 Uniwersytecka Str., 25-406 Kielce, Poland,*
e-mail: agajw@yahoo.com; agnieszka.jablonska-wawrzycka@ujk.edu.pl

[2] *Institute of Biology, Jan Kochanowski University of Kielce, 7 Uniwersytecka Str., 25-406 Kielce, Poland,*

[3] *Institute of Physics, Jan Kochanowski University of Kielce, 7 Uniwersytecka Str., 25-406 Kielce, Poland,*

[4] *Faculty of Environmental, Geomatic and Energy Engineering, Kielce University of Technology, 7 Tysiąclecia Państwa Polskiego Ave., 25-314 Kielce, Poland*

**CONTENTS**

**1. Experimental section ……………………………………………..S2 – S3**

**2. Supporting figures…………………………………………..............S4 – S5**

**3. Supporting table……………………………………….................S6**

## 1. Experimental section

*Syntheses of ruthenium complexes*

*Preparation of [($\eta^6$-p-cymene)$Ru^{II}ClL_1$]$PF_6$ (Ru(II) complex)*

The complex was prepared by reacting 0.5 mmol (0.3066 g) of [($\eta^6$-*p*-cymene)Ru(μ-Cl)Cl]$_2$ with 1 mmol (0.1954 g) of 2-pyridin-2-yl-1*H*-benzimidazole (2,2′-PyBIm) and in the presence of 2.5 mmol (0.4072 g) of $NH_4PF_6$ under refluxing condition in a 15 mL dry solution of $CH_3OH$ for 10 h. Orange crystals of the compound suitable for single crystal X-ray diffraction were obtained by slow evaporation of the solvent at room temperature. The complex was filtered off and dried in a vacuum box. The product was collected in 82% yield. Anal. Calc. (%) for $RuC_{22}H_{23}N_3ClPF_6$: C, 43.25; H, 3.80; N, 6.88; Found: C, 43.34; H, 3.68; N, 6.93. IR ($cm^{-1}$): 3099(br), 3058(br), 2976(ms), 2970(ms), 2876(w), 1610(ms), 1594(m), 1485(s), 1458(s), 1447(s), 1328(ms), 1261(ms), 1150(ms), 1116(ms), 1034(ms), 878(vs), 836(vs), 786(s), 752(vs), 667(s), 558(vs).

*Preparation of mer-[$Ru^{III}Cl_3(CH_3CN)L_1$]·$L_1$·$3H_2O$ (Ru(III) complex)*

Complex *mer*-[$Ru^{III}Cl_3(CH_3CN)L_1$]·$L_1$·$3H_2O$ was synthesized by addition of 2-pyridin-2-yl-1*H*-benzimidazole (0.0976 g, 0.5 mmol) in a mixture of acetonitrile–ethanol to a stirred solution of ruthenium(III) chloride (0.1 M, 5 mL, 0.5 mmol). Then, ethanol and hydrochloric acid were added to this solution (6 : 1 v/v). The reaction mixture was heated at reflux for one hour. Afterwards, the solution was left to crystallize at room temperature. After one week, brown crystals were obtained and dried in a vacuum box. Yield 16%. Anal. Calc. (%) for $RuCl_3C_{26}H_{27}N_7O_3$: C, 45.0; H, 4.1; N, 14.1; Found: C, 45.2; H, 4.6; N, 13.9. IR: 3485(br), 3182(br), 3155(br), 3122(br), 3085(w), 2983(vs), 2925(vs), 2867(vs), 2843(vs), 2339(s), 1645(s), 1610(s), 1571(s), 1475(s), 1453(s), 1143(s), 1155(s), 1117(s), 668(s), 626(s), 614(s).

*Preparation of ($H_2L_1$)$_2$[$Ru^{III}Cl_4(CH_3CN)_2$]$_2$[$Ru^{IV}Cl_4(CH_3CN)_2$]·2Cl·$6H_2O$ (Ru(III/IV) complex)*

This compound was prepared by refluxing 0.1 M $RuCl_3$ (5 mL, 0.5 mmol) and 2,2′-PyBIm (0.0484 g, 0.25 mmol) in acetonitrile–ethanol, hydrochloric acid for 2 hours using the procedure employed for the Ru(III) complex. Only the molar relation of reagents underwent the modification. The solution mixture resulted in the formation of a brown precipitate, which was filtered off and discarded. The filtrate was allowed to evaporate slowly affording orange crystals within two weeks. The product was separated by filtration, and dried under vacuum conditions. The compound was collected in 15% yield. Anal. Calc. (%) for $Ru_3Cl_{14}C_{36}H_{50}N_{12}O_6$: C, 28.0; H, 3.3; N, 10.9; Found: C, 28.4; H, 3.45; N, 11.1. IR: 3442(br), 3212(br), 3068(w), 3042(w), 2975(w), 2920(w), 2835(w), 2295(vs), 1626(s), 1614(s), 1493(vs), 1476(vs), 1453(vs), 1445(vs), 1153(s), 1136(s), 1119(s), 668(ms), 625(ms), 586(ms), 584(ms).

*FT-IR spectroscopic study*

The IR spectrum of the Ru(II) complex exhibits broad absorption bands in the range of 3100–3060 $cm^{-1}$ which are associated with the N-H and C-H aromatic vibrations from the pyridyl-benzimidazole and *p*-cymene molecules. Slight changes in the shift of the $\nu_{C=C}$ and $\nu_{C=N}$ stretches (1610–1440 $cm^{-1}$) vibrations, when compared with the frequencies of the free ligand, suggest that nitrogen atoms are involved in coordination to the metal ion. The presence of new, very strong peaks at 836 $cm^{-1}$ and 558 $cm^{-1}$ is attributed to $\nu_{P\text{-}F}$ stretching modes of the hexafluorophosphate which can act as a counter-ion.

The Ru(III) complex demonstrates a broad band, approximately 3400 $cm^{-1,}$ which was attributed to the $\nu_{O\text{-}H}$ vibration of water molecules. Weak peaks observed in the region of 3200 to 3100 $cm^{-1}$ can be assigned to the $\nu_{N\text{-}H}$ stretching vibrations. The bands at 3085 $cm^{-1}$ correspond to aromatic C-H stretching. The bands in the region 1650–1570 $cm^{-1}$ were attributed to the C=C, C=N stretching modes of the pyridine and imidazole rings. The shift in the $\nu_{C=N}$ vibration to lower energies confirms the coordination of 2,2′-PyBIm to the central ion ($\Delta$ = 13 $cm^{-1}$). An absorption band at 2339 $cm^{-1}$ was attributed to the $\nu_{C\equiv N}$ asymmetric vibration for the Ru(III) complex. The presence of the $Cl^-$ ion is indicated by the bands in the region of 670 to 545 $cm^{-1}$.

In the infrared spectrum of the Ru(III/IV) complex, the broad absorption bands are assigned to the $\nu_{O\text{-}H}$, $\nu_{N\text{-}H}$ and $\nu_{C\text{-}H}$ stretching vibrations from the protonated ligands and water molecules (3450–3030 $cm^{-1}$). It is observed that the spectrum shows a shift in the $\nu_{C=N}$ and $\nu_{C=C}$ stretching vibrations to higher energies when compared with the energies of the free ligand. It indicates a lack of ligand coordination to the ruthenium ion. The IR spectrum of the Ru(III/IV) complex demonstrates a strong, sharp band at 2295 $cm^{-1}$, which confirms the existence of an acetonitrile group in the compound.

The spectroscopic data presented correlate well with the structural parameters presented in [31,32].

*Magnetic measurements*

The low spin $d^6$ configuration of *p*-cymene ruthenium complex was confirmed by magnetic susceptibility measurement at room temperature. This supports the divalent state of ruthenium ion in the complex.

Magnetic susceptibility measurements at room temperature show that the Ru(III) complex is paramagnetic, which supports the trivalent state of ruthenium in this complex. The obtained value of 1.96 $\mu_B$, which corresponds to one unpaired electron, (S=1/2), suggests a low spin $t_{2g}^5$ configuration in an octahedral environment. The $\mu_{eff}$ is in agreement with the spin-only value of 1.73 $\mu_B$ required for the $d^5$ configuration.

The magnetic results are in good agreement with that reported in [31,32].

## 2. Supporting figures

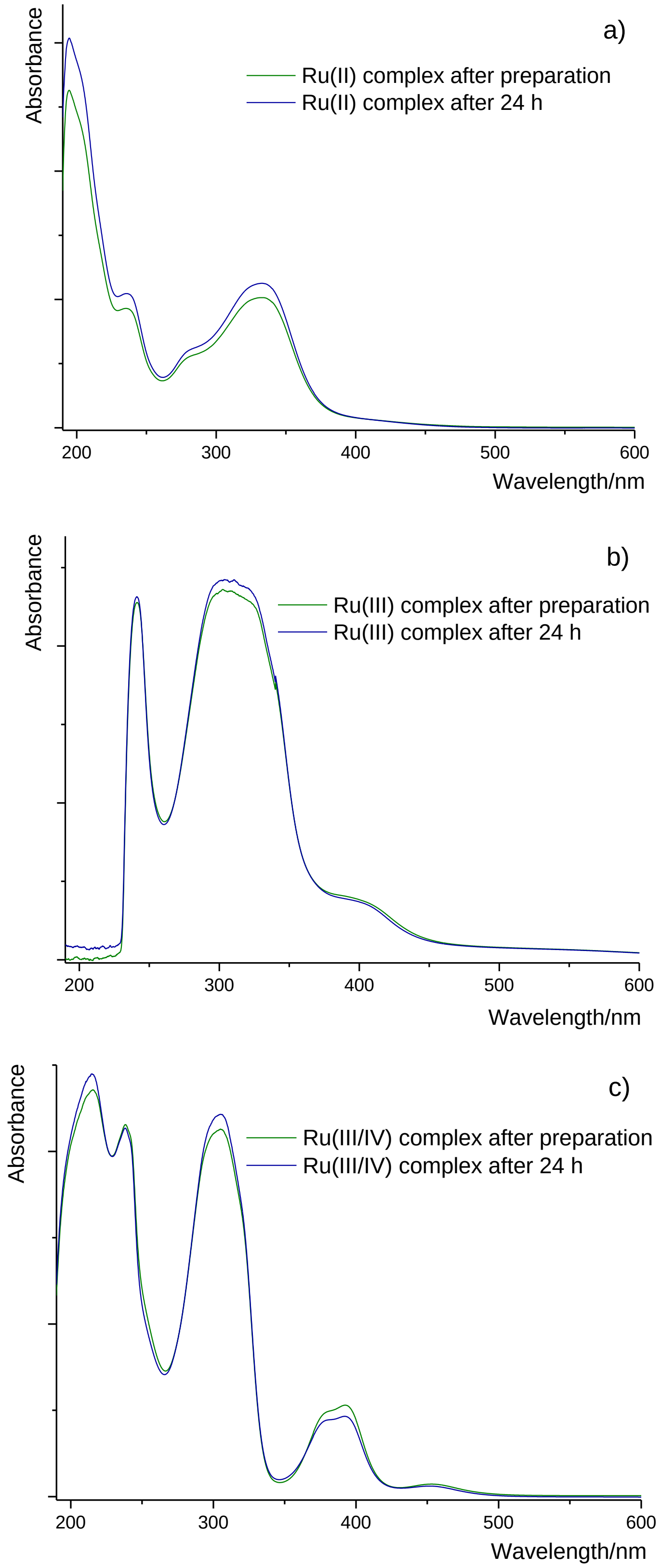


**Figure S1.** Time dependence of UV-Vis spectra of the ruthenium complexes: (a) for the Ru(II) complex (solvent – $H_2O$); (b) for the Ru(III) complex (solvent – $H_2O$/DMSO), and (c) for the Ru(III/IV) complex (solvent – $H_2O$). The concentrations of prepared solutions of the compounds were 1 mM.

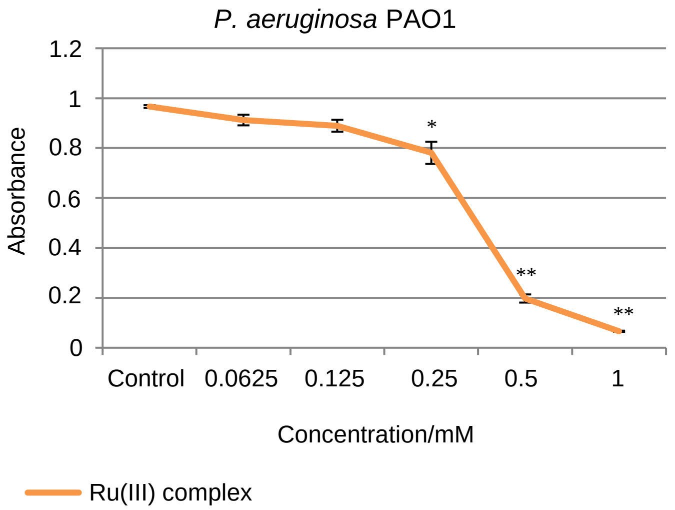


**Figure S2.** The effect of different concentrations of the Ru(III) complex on *P. aeruginosa* PAO1 planktonic cells; * — $p < 0.05$ and ** — $p < 0.001$.

## 3. Supporting table

**Table S1.** Percentage (± standard deviation) of adhesion of *P. aeruginosa* PAO1 in the presence of the Ru complexes over time.

| Time | *P. aeruginosa* PAO1 | $RuCl_3 \cdot xH_2O$ | Ru(II) precursor | 2,2′-PyBIm | Ru(II) complex | Ru(III) complex | Ru(III/IV) complex |
|---|---|---|---|---|---|---|---|
| 1 h | 100 ± 14.64 | 22.68 ± 4.16 | 16.85 ± 2.19 | 36.13 ± 1.00 | 28.49 ± 1.35 | 25.41 ± 5.63 | 9.21 ± 1.14 |
| 2 h | 100 ± 9.19 | 30.72 ± 3.54 | 18.67 ± 1.46 | 35.68 ± 0.99 | 50.10 ± 9.54 | 21.32 ± 3.28 | 6.25 ± 0.62 |
| 3 h | 100 ± 12.91 | 57.82 ± 0.11 | 28.21 ± 1.55 | 55.11 ± 1.71 | 51.35 ± 2.77 | 27.71 ± 6.29 | 5.67 ± 0.37 |
